# Advances in the prevalence and treatment of depression for adolescents: a review

**DOI:** 10.3389/fphar.2025.1574574

**Published:** 2025-05-06

**Authors:** Chunyu Yin, Mengting Xu, Zhiyuan Zong

**Affiliations:** ^1^ Department of Neonatal Medical Center, Children’s Hospital of Nanjing Medical University, Nanjing, China; ^2^ School of Basic Medicine and Clinical Pharmacy, China Pharmaceutical University, Nanjing, China; ^3^ Department of Engineering Science, University of Oxford, Oxford, United Kingdom

**Keywords:** adolescent depression, COVID-19, antidepressants, risk factors, psychotherapy

## Abstract

**Background:**

Depression is a psychological condition in adolescents caused by various factors. Many serious consequences can be associated with depression, such as irritability, emotional instability, and suicide. Meanwhile, the incidence of depression and suicide among adolescents was also affected during the pandemic of COVID-19 in 2019. This phenomenon of adolescent depression should be drawn extensive concern by the community, which affects their physical and mental health.

**Main body:**

This review describes the epidemiology, high-risk factors, and treatment of adolescent depression. The onset of depression is probably attributable to preterm birth, growth environment, genetic. We also identify that the COVID-19 pandemic, initiated in late 2019,affects adolescent mental health. Antidepressants and psychotherapy are conventional treatments for depressive disorders. However, it is controversial whether antidepressants are as effective and safer as psychotherapy, and a combination of the two could provide more benefit to this population than antidepressants alone. We also summarize some antidepressants developed for novel targets. Improving the efficacy and safety of treatment to reduce the suicide rate among adolescents is the primary goal of clinical research. Existing treatment modalities and drugs are not sufficient to achieve clinical demands, so that new therapeutic targets will be promising for such patients.

**Conclusion:**

A variety of factors can contribute to depression in adolescents. Adolescent depression should be mainly treated with non-pharmacological. A combination of guideline-recommended antidepressants should be used if uncontrolled with non-pharmacological, but adverse drug reactions and suicidal ideation should be closely monitored.

## Graphical Abstract

**Figure:**
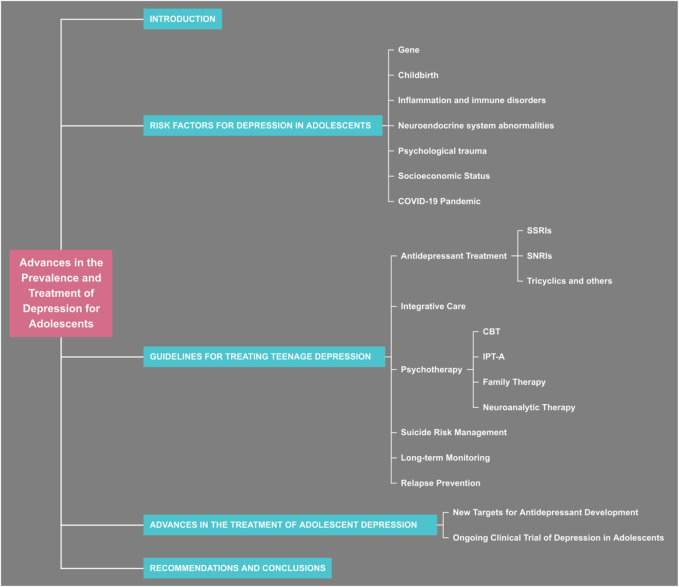
Overview Framework Diagram.

## 1 Introduction

Depression is an emotional disorder with high incidence, high disability rate and high mortality rate, which seriously endangers human mental health. Nowadays, with the great changes in the social environment, the incidence of depression is gradually becoming younger. Globally, one in seven children aged 10–19 experiences a mental disorder, accounting for 13% of the global disease burden in this age group. Depression, anxiety, and behavior disorders are significant causes of illness and disability among adolescents. There was a global increase in the prevalence and incidence associated with adolescent depression between 1990 and 2019. In terms of regional trends, age-standardized rates of prevalence and incidence increased in high-income regions like North America and Australasia, whereas they generally declined in low- and middle-income regions (Yang et al., 2024). A meta-analysis that collected prevalence data on depression or depressive symptoms among children and adolescents aged <18 years included 96 studies published between 1989 and 2022. The pooled prevalence rates for mild to severe depression, moderate to severe depression, and severe depression were 21.3%, 18.9%, and 3.7%, respectively. It is approximately one in five children and adolescents globally experience depression or depressive symptoms, and this proportion is increasing over time (Lu et al., 2024). The consequences of failing to address adolescent mental health can extend into adulthood, which can impair physical and mental health and limit opportunities to lead a fulfilling life in adulthood. A 2012–2015 epidemiological survey showed an increase in depression and suicide rates, especially among female adolescents (Lu et al., 2021). In this age group, depression is associated with a variety of socio-demographic, gene, premature, and early inflammation (Mridha et al., 2021). This review summarizes the epidemiology, risk factors, and current clinical medications for depression in adolescents.

Adolescence is a vital period for physical and mental development, and depression during this time can have more profound long-term impacts. It may result in disrupted education, weakened social skills, and even affect career prospects and quality of life in adulthood. During the pandemic, changes in learning methods (e.g., online learning), social isolation, and uncertainties about the future have made adolescents more susceptible to psychological stress. Compared with the elderly, intervention strategies for adolescent depression can be more effectively carried out through schools and communities. Additionally, adolescents are often found in concentrated settings like schools, which makes large-scale surveys and psychological assessments more feasible, allowing for the collection of more comprehensive and accurate data. Thus, it is very necessary to focus on the adolescent group to reduce the incidence of depression.

Corona Virus Disease 2019 (COVID-19), which is the more serious virus, was subsequent global epidemic since 2019 have seen a siginificantly upward trend in the incidence of adolescent depression in many countries, including China. Research on the effects of the COVID-19 pandemic on adolescent depression indicates that the pandemic has significantly affected adolescent depression, with prevalence and incidence rates in 2020 and 2021 far surpassing predictions. The COVID-19 pandemic has notably increased the burden of depression and worsened health inequalities (Zhu et al., 2025). Simultaneous, the incidence of anxiety disorders jumped from 720.26 per 100,000 in the pre-pandemic period (2018–2019) to 880.87 per 100,000 during the pandemic (2020–2021). Likewise, the incidence of major depressive disorder (MDD) climbed from 2,333.91 per 100,000 to 3,030.49 per 100,000. This trend was more evident in high-income countries (HICs). Environmental factors such as increased social anxiety, school closures, economic instability, and racial tensions have negatively impacted adolescent mental health (Kim S. et al., 2024). Additionally, A global survey indicated that COVID-19 impact indicators, especially the daily SARS-CoV-2 infection rate and reduced human activity, were associated with an increase in the prevalence of major depressive disorder among adolescents (Zhou et al., 2025).The pandemic has not only intensified existing risk factors for depression but has also created new ones, such as prolonged periods of social isolation and increased exposure to family stress and economic hardship (Du et al., 2025).

A variety of nonpharmacologic, pharmacologic, and combination treatment options are available for adolescent depression, and many interventions have insufficient clinical evidence and are more controversial in terms of efficacy and side effects (Viswanathan et al., 2020). Different levels of depression may require different interventions. For some treatment-resistant or severe depression, antidepressants are required. A study from 2007 to 2017 showed a dramatic increase in the proportion of antidepressant users, especially among 15–19-year-old (Wesselhoeft et al., 2020). However, since possible side effects of antidepressants, such as venlafaxine, may increase the risk of suicide-related behaviors, psychotherapy is widely used in this population, albeit with limited efficacy. At the same time, there are a wide variety of antidepressants and clinical abuse, so their efficacy and safety should be clarified (Evans and Sullivan, 2014). Another unfortunate critical aspect is that current treatments are inadequate to fulfill clinical needs. In the context of increasing morbidity yearly, we urgently seek more effective interventions to deal with clinical stress.

Therefore, this study aims to illustrate how the COVID-19 pandemic has affected adolescent mental health and its link to adolescent depression. The outbreak and control measures of COVID-19 have significantly disrupted the daily lives, education, and social interactions of children and adolescents, subsequently affecting their mental wellbeing. Understanding these impacts can help devise proactive strategies for future public health emergencies to safeguard the mental health of young people. Furthermore, integrating insights from prior studies and guidelines, along with the main causes and effective treatments for adolescent depression, we have outlined the latest treatment objectives and directions to provide a reference for future therapeutic strategies.

## 2 Risk factors for depression in adolescents

### 2.1 Gene

Previous research indicates that genes like sirtuin1(SIRT1), 5-hydroxytryptamine (5-HT), Brain-derived neurotrophic factor (BDNF), Catechol-O-methyltransferase (COMT), oxytocin receptor(OXTR), nuclear receptor subfamily three group C member 2(NR3C2), solute Carrier Family 6 Member 4(SLC6A4), and FKBP Prolyl Isomerase 5(FKBP5) are associated with depression.

#### 2.1.1 Sirtuin1 (SIRT1)

In 2014, Flint, Kendler, and a collaborative team completed a DNA sequence analysis of 5,303 Han Chinese women with major depression, along with 5,337 control sequences. The analysis identified two depression-associated mutation sites on chromosome 10, one mutation located near Sirtuin1(SIRT1) and another in the intron of the phosphorlysine phosphohistidine inorganic pyrophosphate phosphatase (LHPP) gene, encoding a protease whose function is currently incompletely understood. SIRT1 gene is an important protease in mitochondria (Converge, 2015). SIRT1 regulates neuronal transmission-related genes in D1-MSNs, affecting electrophysiological, morphological, and behavioral endpoints. It suggests that it may influence synaptic transmission gene expression in brain reward regions linked to anxiety and depression-like behaviors (Kim H. D. et al., 2024). A study on major depressive disorder risk shows that LHPP gene deletion in the brain or local knockout in mouse prefrontal cortex increases depression-like behaviors under chronic social defeat stress (Lin et al., 2023).

#### 2.1.2 5-Hydroxytryptamine (5-HT)

The 5-HT system is one of the most extensively studied neurotransmitter systems to date. Downregulation of 5-HT in the human brain plays a critical role in the pathogenesis of affective psychiatric disorders. Notably, compared to adult depression, behavioral and social emotional problems are more prominently manifested in adolescent depressive disorders. 5-HT downregulation is crucial in the development of affective disorders and is a key factor in adolescent depression. In a study of Mexican adolescents, the S allele and S/S genotype of the 5-HTT gene-linked polymorphic region(5-HTTLPR) polymorphism were associated with depression and a history of suicide attempts (Sarmiento-Hernández et al., 2019). In Chinese adolescents, rare variants in the 5-HTTLPR gene may increase the risk of major depressive disorder with suicidal ideation (Liu et al., 2020). Considering gene-environment interactions, childhood abuse and 5-HTTLPR gene polymorphisms interact in adolescent depression development, with 5-HTTLPR moderating the link between childhood abuse and adolescent depression (Rocha et al., 2015).

#### 2.1.3 Brain-derived neurotrophic factor (BDNF)

BDNF is a critical regulator of early neuronal development and survival. BDNF plays a pivotal role in neurodevelopment, synaptogenesis, and synaptic plasticity. Val66Met (rs6265) represents a well-characterized single-nucleotide polymorphism (SNP) in the BDNF gene. Emerging evidence indicates that the Met allele exhibits stronger associations with amygdala-cortical connectivity patterns linked to depression, particularly in adolescent females (Wheeler et al., 2018). The Val allele appears to influence the development of early-adolescent depressive disorders, with Val/Val and Val/Met genotypes being predominant in female adolescents diagnosed with depressive disorders (Hilt et al., 2007).

#### 2.1.4 Catechol-O-methyltransferase (COMT)

COMT is a dopamine-degrading enzyme that plays a crucial role in the metabolism of dopamine, norepinephrine, and epinephrine in the prefrontal cortex (PFC). The interaction between COMT genotypes and life events has been noted. Among 4-5-year-old children with the Val/Val genotype, those who have experienced severe life events are more likely to exhibit aggressive behavior, which indicates the significance of gene-environment interactions in psychiatric-related behaviors (Hygen et al., 2015). Additionally, adolescents with at least one Met allele genotype are more susceptible to the influence of early-life adversity and tend to display symptoms of hyperactivity and impulsivity (Abraham et al., 2020).

#### 2.1.5 Oxytocin receptor(OXTR)

The oxytocin system is involved in social behavior, emotional regulation, and stress response. Polymorphisms in the OXTR gene have been linked to adolescent depression, mainly through their effects on social stress sensitivity and emotional regulation.Thompson et al. found that the interaction between the OXTR rs2254298 polymorphism and maternal history of depression could significantly increase depressive and anxiety symptoms in adolescent girls. Specifically, girls carrying the AG genotype showed higher levels of depression in high - family adversity environments (van Roekel et al., 2013). Another study by van Roekel et al. using the experience - sampling method reported that the A allele of OXTR rs53576 was significantly associated with the feeling of loneliness in adolescent girls, especially in negative social situations. These findings suggest that OXTR polymorphisms may increase the vulnerability of adolescents to depression through their impact on social and emotional processes (Thompson et al., 2011).

#### 2.1.6 Nuclear receptor subfamily 3 group C member 2(NR3C2)

The NR3C2 gene encodes the mineralocorticoid receptor, which is involved in the regulation of the body’s stress response.Vinkers et al. reported that NR3C2 haplotypes showed sex-dependent moderation of depression susceptibility following childhood maltreatment. The CA haplotype of NR3C2 had different effects on males and females. In females, CA carriers had a lower risk of depression in the context of childhood abuse, while in males, CA carriers had a higher risk. This indicates that the NR3C2 gene may interact with early - life stress to influence the development of adolescent depression in a sex - specific manner (Vinkers et al., 2015).

#### 2.1.7 Solute carrier family 6 member 4(SLC6A4) and FKBP Prolyl Isomerase 5(FKBP5)

In addition, other studies have shown that the SLC6A4 (Serotonin Transporter Gene) and FKBP5 (FK506-Binding Protein 5 Gene) are also associated with adolescent depression.The SLC6A4 gene encodes the serotonin transporter, which plays a key role in the regulation of serotonin levels in the brain. Serotonin is an important neurotransmitter involved in mood regulation. Liu et al. reported that rare variants in SLC6A4 were associated with major depressive disorder with suicidal ideation in Han Chinese adolescents and young adults[17]. The FKBP5 gene is involved in the regulation of the hypothalamic-pituitary-adrenal (HPA) axis, which is a major stress - response system in the body.Polymorphisms rs1360780 and rs3800373 in the FKBP5 gene were associated with suicidal events in adolescents with treatment-resistant depression (Brent et al., 2010).

Previous sections have highlighted that gene-environment interactions collectively influence adolescent mental health. Apparently, gene-environment interactions play an important role in regulating the risk of depression. Functional 43bp insertion/deletion polymorphism in the promoter region of the 5-HT transporter gene, which was shown to interact with stressful events, particularly child abuse, contributing to the diagnosis of depression and predicting suicidal ideation (Caspi et al., 2003). Gene-environment interactions are a causative mechanism involved in promoting the onset of depression by influencing the patient’s susceptibility to the environment (Uher, 2014). Currently, several environmental factors have been identified to promote depression susceptibility, including intrauterine infections, nutritional deficiencies, maternal depression, perinatal complications, social disadvantage, minority status, child abuse, bullying, traumatic events, and marijuana use. These factors, especially those stressful experiences that happen early in lifespan have a significant influence on the development of the brain (Heim and Binder, 2012), (Booij et al., 2014).

#### 2.1.8 Childbirth

The impact of delivery time and birth weight on long-term physical and mental health risks warrants further investigation. With advances in healthcare, the mortality rate of preterm infants has decreased significantly compared with the past. Preterm infants are babies born before 37 weeks. Meanwhile, prematurity and low birth weight (PT/LBW) are the two major contributors to neurocognitive deficits and developmental delays in children. As early as 2004, a prospective cohort study of 2032 adolescents found that the prevalence of depression among PT/LBW patients had 11-fold higher compared with regular participants (95% CI 2–62). The cumulative rate of depression over 30 months was 15.2% (95% CI 11.1–20.5) in PT/LBW compared with 1.8% (95% CI 1.6–2.1) in those standard deliveries (Patton et al., 2004). A meta-analysis was performed to investigate the proportion of PT/LBW patients aged 10–25 years diagnosed with depression and anxiety disorders. A total of five studies met the inclusion criteria, and patients with PT/LBW had a higher risk of developing anxiety and depressive symptoms compared with controls [3.66, 95% CI: 2.57–5.21; or 2.86, 95% CI: 1.73–4.73] (Burnett et al., 2011).

On the other hand, recent studies have showed that different modes of delivery can impact the mental symptoms of adolescents. A population-based registry study in Finland included 22,181 children and adolescents with anxiety disorders and 74,726 controls. The study considered mode of delivery, Apgar score, umbilical artery pH, and neonatal monitoring as exposure variables for anxiety disorders and specific anxiety disorders. Both unplanned and planned cesarean sections were associated with an increased likelihood of anxiety disorders in children and adolescents. Planned cesarean sections and the need for neonatal monitoring increased the odds for specific phobia (Ståhlberg et al., 2022). In a national cohort study, participants diagnosed with bipolar disorder (BPD) between the ages of 13 and 34 were followed to examine the relationship between neonatal characteristics and pregnancy complications and the risk of BPD in offspring. The findings revealed that very preterm birth/extremely preterm birth (22–31 weeks) was associated with higher hazard ratios (HRs) for BPD. Cesarean section, compared with vaginal delivery, was associated with a 1.2-fold and 1.6-fold increased risk of BPD, respectively (Beer et al., 2023).

### 2.2 Inflammation and immune disorders

Depression may be associated with early inflammatory response, with studies showing a decreased synaptic density and increased inflammation in the anterior cingulate cortex (ACC) in the brains of depressed patients. Because early inflammation may cause an increased susceptibility of microglia in the ACC to random stressful events during pubertal development (Zheng et al., 2021). One study included 11,786 children with infections and used infection severity (“low” = 0–4 infections, “moderate” = 5–6, “high” = 7-9, or “very high” = 10–22 infections) as an additional analysis of exposure. A common early childhood infection was found to be associated with depressive symptoms in mid-adolescence and subsequent psychotic experiences in childhood but not in early adulthood (Chaplin et al., 2020).

Depression is associated with increased levels of pro-inflammatory cytokines such as IL-6 and TNF-α. These cytokines can induce indoleamine 2,3-dioxygenase (IDO) activation, leading to the diversion of tryptophan metabolism from 5-HT synthesis to the production of neurotoxic kynurenine, thereby contributing to the development and exacerbation of depression (Bernardoni et al., 2024). Microglia, the immune cells of the central nervous system, play a crucial role in maintaining neural homeostasis. However, excessive activation of microglia can result in the overproduction of inflammatory factors, excessive synaptic pruning, and increased cell migration, which may trigger and accelerate neuronal damage in various neurological disorders, including depression. Studies have shown that the transcription factor MEF2C can restrain microglial overactivation by inhibiting CDK2 activation, thus protecting against neuroinflammation and associated neurological disorders (Hu et al., 2025).

The relationship between gut microbiota and depression has been a hot topic in recent research. Studies have shown that gut microbiota dysbiosis is closely related to the onset and progression of depression. This article systematically reviews the recent research on the ‘microbiota-gut-brain axis’ (MGBA), exploring the role and mechanisms of gut microbiota in preclinical and clinical studies. Additionally, it discusses the potential of modulating gut microbiota as a promising therapeutic approach for depression and suggests future research directions, aiming to provide a theoretical basis for research and clinical management of depression (Zhang et al., 2025). The mechanisms by which gut microbiota can impact the pathophysiology of depression are multifaceted. They include influencing tryptophan metabolism, modulating the hypothalamic-pituitary-adrenal (HPA) axis, and affecting brain-derived neurotrophic factor (BDNF). The gut microbiota plays a key role in determining the fate and activity of tryptophan, which is a crucial factor in mental health management. When the metabolism of tryptophan is activated along the kynurenine pathway, it can lead to an increase in the production of neurotoxic quinolinic acid, which may have adverse effects on mental health (Clerici et al., 2025).

### 2.3 Neuroendocrine system abnormalities

Neurotransmitter imbalance is a significant factor in the pathophysiology of depression. Studies have shown that three neurotransmitters,5-HT, dopamine (DA), and noradrenalin (NE) have a significant impact on the brain circuits involved in motivation, emotion regulation, cognitive performance, and psychological stress responses of MDD. The monoamine hypothesis posits that 5-HT deficiency is one of the recognized mechanisms associated with the development of depression. Additionally, glutamic acid (GLU) and γ-aminobutyric acid (GABA) serve as excitatory and inhibitory neurotransmitters in the GABA transmission system respectively. The abnormal GABA pathway caused by the metabolic imbalance between both may also participate in the initiation of MDD (Zhao et al., 2024).

Hormonal dysregulation, particularly in the hypothalamic-pituitary-adrenal (HPA) axis, is also linked to depression. Blunted neuroendocrine stress responses have been found in youth with MDD across different neuroendocrine systems and in both females and males with MDD. This suggests that the HPA axis may be dysregulated in individuals with depression, leading to maladaptation to stress stimulation (Bernhard et al., 2025).

### 2.4 Psychological trauma

Psychological stress is a significant factor contributing to adolescent depression. Studies have shown that adolescents experiencing high levels of academic stress are more likely to develop depressive symptoms. This stress can stem from heavy academic loads, strained peer and teacher-student relationships, and high parental expectations, all of which can negatively impact adolescents’ mental health and future outlook on life.The interplay between anxiety and depressive symptoms is also closely linked to psychological stress. Adolescents with depression often exhibit heightened levels of anxiety, which can exacerbate depressive symptoms. Research indicates that hope levels and coping strategies mediate the relationship between anxiety and depressive symptoms, with hope levels playing a more significant role than coping strategies. This suggests that fostering a sense of hope and promoting positive coping mechanisms can help alleviate depressive symptoms in adolescents.Moreover, psychological stress can affect adolescents’ emotional clarity and overall outlook on life. A nurturing and supportive environment, both at school and at home, can significantly enhance adolescents’ sense of hope and coping abilities, while a lack of emotional support can lead to increased psychological stress and depressive symptoms (Lin et al., 2024).

### 2.5 Socioeconomic status (SES)

SES has a significant impact on adolescent depression. Families with lower SES often face financial strain, limited access to quality education and healthcare, and higher levels of stress, all of which can contribute to the development of depressive symptoms in adolescents. Additionally, adolescents from lower SES backgrounds may experience more exposure to adverse life events and neighborhood violence, further exacerbating their risk for depression. The relationship between SES and adolescent depression is also influenced by the quality of schools and educational resources. Schools in low-SES areas often have fewer resources, less qualified teachers, and higher student-teacher ratios, which can negatively affect students’ academic performance and mental health. Moreover, the stress associated with academic underachievement can lead to feelings of hopelessness and depression among adolescents (Fortenberry, 2003).

In HICs, adolescents faced substantial social, academic, and familial stressors during the pandemic, including challenges in adapting to online education, social isolation, and increased financial strain within families. These factors exacerbated mental health issues, despite the availability of robust healthcare systems and mental health services.In contrast, low- and middle-income countries experienced even greater challenges due to limited healthcare resources and pre-existing health inequities. School closures, social isolation, and heightened economic pressures created additional barriers for adolescents seeking mental health support. Moreover, cultural stigma surrounding mental health in many low-income countries often discouraged adolescents from seeking help, further hindering diagnosis and treatment (Zhu et al., 2025).

The pandemic has highlighted global disparities in healthcare resource distribution, particularly in low- and middle-income countries where COVID-19 intensified pre-existing health inequalities. While high-income countries faced significant challenges, they had greater capacity for implementing mental health interventions, education, and resource allocation. However, low-income countries faced substantial deficits in these areas, leaving their adolescent populations more vulnerable to the pandemic’s adverse effects.

### 2.6 COVID-19 pandemic

COVID-19 makes a huge impact on adolescents and their families around the world (Fegert et al., 2020; Loades et al., 2020). According to a scientific brief published by the World Health Organization (WHO) in 2022, there has been a dramatic 25% increase in the incidence of anxiety and depression [(World Health Organiztionb)]. The presentation provides a comprehensive evaluation of the impact of COVID-19 on mental health and mental health services, revealing that the epidemic is affecting the mental health of young people, who are at increased risk for suicide and self-harm. The COVID-19 epidemic is also influencing mental health, suicide rates, and mental health emergency department rates (Meade, 2021; Czeisler et al., 2020; Manzar et al., 2021). COVID-19 has impacted mental health for several reasons. The most important reasons may be lockdown and school closures, limited opportunities for physical activity, social gatherings and interaction with peers, uncertainty regarding the consequences of COVID infection, closed or limited outpatient services, reluctance to consult and fear of being infected in the early stages of the pandemic (Michaud et al., 2022). Long-term psychological consequences of COVID-19 were also observed in specific populations. A multinational study found that individuals with COVID-19 had a significantly higher risk of developing depression, anxiety disorders, and sleep disorders within the first 6 months post-infection. While these risks decreased over time, elevated rates of depression and anxiety persisted in some countries during the medium- and long-term follow-up periods. Furthermore, the pandemic-related psychosocial stressors, such as social isolation and financial insecurity, contributed to the overall mental health burden (Chai et al., 2025).

A study reviewed visit rates between January to October on 2019 and 2020 and discovered that from 2019 to 2020, the rate of such visits increased by 24% for children aged five to 11 (from 783 visits per 100,000 to 972 visits per 100,000), while the rate of mental health-related visits among aged 12 to 17 increased by 31%. These increases likely reflect the increased distress of adolescents due to pandemic-related stress (Leeb et al., 2020). One systematic review across 16 studies from 2019 to 2021 showed (n = 40,076) that globally, adolescents from diverse backgrounds experienced more anxiety, depression, and stress as a result of the COVID-19 pandemic (Jones et al., 2021). Another Chinese cross-sectional survey including 4,342 adolescents found that anxiety, depression, and stress were prevalent among adolescents experiencing family isolation and school closure. High school students are more seriously affected by the epidemic, and the efficiency of taking online classes is significantly reduced. Due to the high academic pressure, they are at greater risk of depression and anxiety (Tang et al., 2021).

Adults are the dominant population affected by COVID-19. The pediatric population is less affected by COVID-19. Most published reports from different countries indicate that pediatric patients account for less than 2% of reported cases. However, due to the large number of infections globally, this 2% is also a large population. According to the WHO report, approximately 10%–20% of people who recover from the initial illness experience various long-term effects, with depression and anxiety being one of the more visible sequelae of COVID-19 [(World Health Organiztionc)].

There is an urgent need for effective treatments to treat the various depressive symptoms caused by COVID-19. To this end, multiple clinical trials for depression in adolescents affected by COVID-19 are planned or conducted. Tables 1, 2 list the clinical trials and studies for adolescent depression since the outbreak of COVID-19. Several studies have shown that anxiety and depression are more prevalent during the COVID-19 pandemic, especially among Chinese adolescents. The investigation found that girls were more likely to be anxious and depressed. However, the rise was not limited to depression; suicide rates also had increased during COVID-19, particularly among girls (Patton et al., 2004; Burnett et al., 2011; Ståhlberg et al., 2022; Beer et al., 2023; Zheng et al., 2021; Chaplin et al., 2020; Bernardoni et al., 2024).

**TABLE 1:** Clinical trials on adolescents depression under the COVID-19.

Identifier	Study title	Interventional	Population	Study objective
NCT04524598	A CBT-based Mobile Intervention as First-Line Treatment for Adolescent Depression During COVID-19	CBT-based intervention *vs* psycho-education	Depressed teenagers aged 13–21(227)	Assessing digital therapy based on CBT versus psycho-education for clinical effectiveness and safety
NCT04634903	Testing Scalable, Single-Session Interventions for Adolescent Depression in the Context of COVID-19	ST-SSI *vs* BA-SSI *vs* GM-SSI	Depressed teenagers aged 13–16(2452)	Testing the advantages of each SSI relative to the control group
NCT04780152	TDCS in Pediatric and Teenage Patients with Major Depressive Disorder During COVID-19 Pandemic	Transcranial Direct Current Stimulatio + Fluoxetine *vs* Placebo-simulation + Fluoxetine	Major depressed teenagers aged 10–17(172)	Evaluating the safety and efficacy of nodal transcranial direct current stimulation in children and adolescents with the major depressive disorder during the COVID-19 pandemic
NCT04406558	Psychological Impact of the Health Measures Generated by the COVID-19 in Adolescents	An online questionnaire on the experience of the period of confinement and deconfinement will be sent to the cohort of adolescents followed at the CHI Creteil for a chronic disease or not	Teenagers aged 11–18 with Psychological disorders (400)	To examine the impact of COVID-19 on adolescent mental health
NCT04548544	A Randomized Controlled Feasibility Study of Emotional Wellbeing of Adolescents Undergoing a Mindfulness Training During COVID-19	Training for Awareness, Resilience, and Action	Teenagers aged 14–18(21)	Testing the feasibility of TARA in improving the emotional health of adolescents aged 14–18 years during pandemic COVID-19

Abbreviations: SSI, each single-session intervention; ST-SSI, Supportive Therapy SSI; BA-SSI, Behavioral Activation SSI; GM-SSI, Growth Mindset SSI; CBT, cognitive behavioral therapy; TARA, training for awareness resilience and action.

**TABLE 2:** Research on adolescents depression under the COVID-19.

Author	Country (collection dates)	Population	Research Typology	Primary findings
Stephanie L Mayne et al., (2021)	United States (2019.06–12, 2020.06–12)	Youth 12–21 years old	Cross-sectional analysis	During the pandemic, outpatient depression screening in primary care clinics decreased from 77.6% to 75.8%. The confirmed depression in adolescents increased from 5.0% to 6.2%
Chen et al., (2022)	China (2019.12–2021.05)	Chinese adolescents(N = 41729)	Meta-Analysis	Depressive symptoms were widespread among Chinese adolescents during COVID-19, especially among senior students
Cai et al., (2022)	China (2020.08–2021.03)	COVID-19 high school students	Network analysis	Sadness, irritability, and excessive worry are common emotions, and guilt and suicidal ideation may turn anxiety into depression
Hawes et al., (2021)	United States (2020.03–2020.05)	Adolescents and young adults (N = 451)	Longitudinal studies	In the early years of the COVID-19 pandemic in the United States, depression and anxiety symptoms increased in adolescents, especially females
Chen et al., (2021)	China (2020.02–2020.04)	Adolescents (N = 9554,N = 3886)	Comparison of two cross-sectional studies	Depression and anxiety have increased significantly in Chinese adolescents after the first outbreak. Gender and entering a higher school are positively associated with depression
Magson et al., (2021)	Australia	Adolescents (N = 278)	Longitudinal studies	Significant increase in depressive symptoms and decreased life satisfaction among adolescents, particularly among girls
Shoshani and Kor, (2020)	Israeli (2020.05–2020.07)	Adolescents(N = 1537)	Cross-sectional analysis	Depression and panic symptoms increased significantly during the pandemic. With increased time spent on electronic games, the internet and TV, positive emotions and satisfaction with life decreasing

## 3 Guidelines for treating teenage depression

Numerous antidepressant drugs and treatment modalities are developing, and some of the available interventions can be learned from previous studies. According to the latest guidelines [(Cheung et al., 2018), (National Institute for Health and Care Excellence)], there are three main types of treatment for depression in adolescents: (1) Antidepressant treatment; (2) Psychotherapy interventions; (3) Nonspecific psychosocial interventions in pediatric PC (integrated behavioral health and collaborative care models), which can improve the symptoms of depression in adolescents. Guidelines are targeted for adolescents and include the following key recommendations (For a detailed description, see Table 3):

(1) Active Monitoring: For adolescents with mild depression, active monitoring is suggested as an initial approach. This involves regular follow-ups to assess symptom changes and functional status.
(2) Evidence-Based Treatment: For moderate to severe depression, the guidelines recommend using evidence-based medications and psychotherapeutic approaches. This includes selecting appropriate antidepressants and psychotherapies based on the severity of symptoms and individual patient needs.
(3) Side Effect Monitoring: Close monitoring of potential side effects of medications is essential. This involves regular assessments to ensure the safety and wellbeing of the adolescent during treatment.
(4) Consultation and Co-Management: Primary care clinicians are encouraged to consult with and co-manage care alongside mental health specialists, especially in complex cases or when treatment response is suboptimal.
(5) Outcome Tracking: Ongoing tracking of treatment outcomes is necessary to evaluate the effectiveness of interventions and make necessary adjustments to the treatment plan.
(6) Addressing Partial or No Improvement: Specific steps are outlined for situations where adolescents show partial or no improvement after initial treatment. This includes re-evaluating the diagnosis, considering alternative treatments, and adjusting the management plan accordingly.

**TABLE 3:** Guideline-recommended treatment and management of depression in adolescents.

NICE Guideline: Depression in adolescents and young people (Zhang et al., 2025)			
Severity	5–11-year-olds		12–18-year-olds
Mild	Recommendations for interventions effective of 12–18 year olds are available for 5–11		Digital CBT or group therapy (CBT, NDST or IPT)
Moderate and Severe	Recommend therapies with established evidence of efficacy, such as family therapy, family-based IPT and psychodynamic psychotherapy) and CBT		Individual CBT, IPT-A and family therapy
Guidelines for Adolescent Depression in Primary Care: Clinical Management[38]			
Severity	Initial treatment	If improved	If persistent/not improved
Mild	Active treatment and follow-up over 6–8 weeks (once/twice week)^a^	1. Medication continued for 1 year after complete remission of symptoms 2. To continue monitoring over 24–120 weeks for periodic follow-up, regardless of referral to a mental health professional 3. If continuing with this treatment, keep in contact with a mental health specialist	Reference moderate depression
Moderate	Consulting a psychologist and developing a management plan^a^ ^,^(Lu et al., 2024) 1. Initiating treatment with evidence-based antidepressants and/or psychotherapy in primary care^a^ 2. Monitoring symptoms and adverse events(World Health Organiztiona) 3. Continue mental health counselling ideally	Partially Improved 1. Add antidepressants if not already using on; increase dose to maximum if already using on; add counselling treatment if not already on it 2.Offer further treatment and keep monitoring^a^ Improved Refer to mild depression	1. Re-evaluation of the diagnosis 2. Add antidepressants if not already using on; increase dose to maximum if already using on; add counselling treatment if not already on it 3. Offer further treatment and keep monitoring^a^
Severe	Refer to moderate depression	Refer to moderate depression	Refer to moderate depression

^a^ Psycho-education, supportive counselling and periodic monitoring for depressive symptoms and suicidal tendencies.2. Negotiate roles and/or responsibilities between PC, and mental health and designate case coordination responsibilities. Continue to monitor in PC, after referral and maintain contact with mental health.3. Clinicians need to monitor closely for changes in symptoms and the occurrence of adverse events, like increased suicidal ideation, agitation or mania.

Abbreviations: CBT, cognitive-behavioural therapy; IPT, interpersonal psychotherapy; IPT A, IPT, for adolescents; NDST, non-directive supportive therapy.

### 3.1 Antidepressant treatment

The variable efficacy of antidepressants poses challenges, particularly for patients with treatment-resistant depression (TRD) in clinical. Conventional antidepressants usually have slow onset of action, which takes several weeks to months, can lead to early discontinuation due to lack of confidence in the effectiveness of treatment. Simultaneous, antidepressants are not the preferred treatment modality for adolescents due to their controversial efficacy and tolerability. The antidepressants for adolescents can be divided into four major categories: Selective Serotonin Reuptake Inhibitors (SSRIs), Serotonin Norepinephrine Reuptake Inhibitors (SNRIs), tricyclics and others. Table 4 lists the categories of antidepressants, concluding the effectiveness based on depression scores compared to placebo and recommendations from various studies in adolescents.

**TABLE 4:** List of antidepressant efficacy and risks.

Category (Change as Tracy, 2022)	Drug	MD (95% CI,VS placebo)	Recommendations
SSRIs	Citalopram (Celexa)	−2.90 [-8.23; 2.43] (Base on CDRS-R) [(Hetrick et al., 2021)]	FDA approves fluoxetine and escitalopram for the treatment of depression in adolescents[(Selph and McDonagh, 2019)] SSRIs not recommended as the treatment of choice for mild to moderate depression in adolescents. Consider SSRIs when there is no response for psychological intervention[(Loades et al., 2020),^48^]
	Escitalopram (Lexapro)	−2.62 [-5.29; 0.04] (Base on CDRS-R)[63]	
	Fluoxetine (Prozac, Prozac Weekly, Selfemra, Sarafem)	−2.84 [-4.12;-1.56] (Base on CDRS-R)[63]	
	Fluvoxamine (Faverin, Luvox, Luvox CR)	22.5 ± 5.31(Base on BDI score)[(Apter et al., 1994)]	
	Paroxetine (Paxil, Paxil CR, Pexeva)	−1.43 [-3.90; 1.04] (Base on CDRS-R)[63]	
	Sertraline (Zoloft)	−3.51 [-6.99;-0.04] (Base on CDRS-R)[63]	
	Vilazodone (Viibryd)	−0.84 [-3.03; 3.36] (Base on CDRS-R)[63]	There is insufficient evidence to confirm the efficacy of vilazodone in adolescents with MDD, but safety and tolerance is well [(Durgam et al., 2018b)]
SNRIs	Desvenlafaxine (Pristiq)	−0.07 [-3.57; 3.36] (Base on CDRS-R)[63]	Not recommended for SNRI as primary treatment for adolescent depression. The adverse events that occur in treatment involve physical and emotional/behavioural side effects [(Elbe et al., 2014)]. Venlafaxine is associated with a higher risk of suicide
	Duloxetine (Cymbalta)	−2.70 [-5.03;-0.37] (Base on CDRS-R)[63]	
	Milnacipran (Savella)	−0.38 [-3.41; 2.64][(Fluvoxamine Maleate in the Treatment)]	
	Venlafaxine (Effexor, Effexor XR)	−1.90 [-5.17; 1.37] (Base on CDRS-R)[63]	
Tricyclics	Amitriptyline (Elavil, Endep, Levate), Amoxapine (Asendin), Imipramine (Tofranil, Tofranil-PM), Doxepin (Adapin, Silenor, Sinequan), Maprotiline (Ludiomil), Clomipramine (Anafranil), Desipramine (Norpramin, Pertofrane), Nortryptyline (Aventyl, Pamelor), Protriptyline (Vivactil), Trimipramine (Surmontil, Trimip, Tripramine)	Total: 0.32 [-0.59;-0.04] Adolescents: 0.45 [-0.83;-0.007] Child: 0.15 [-0.32; 0.64] (Base on CDRS-R)[(Leeb et al., 2020)]	Tricyclic drugs are ineffective in adolescent depression and cause more vertigo, upright hypotension, tremors and dry mouth [(Leeb et al., 2020)]
Others	Bupropion (Alpenzin, Budeprion SR, Budeprion XL, Buproban, Wellbutrin, Wellbutrin SR, Wellbutrin XR, Zyban)	Fifty-eight subjects (45.7%) were determined to be responders at 12 weeks (defined by a CGI-Depression-I score ≤2)	Bupropion is effective and well tolerated in adolescents suffering from depression. During the treatment, side effects such as, insomnia, loss of appetite, anxiety, headache, drowsiness, nausea/vomiting could occur [(Jones et al., 2021)]
	Mirtazapine (Remeron, RemeronSolTab)	−2.79 [-6.72; 1.14] (Base on CDRS-R)[63]	Mirtazapine is effective and tolerable in adolescents with MDD [(Mayne et al., 2021)]
	St. John’s Wort	/	Recommended for adult depression [58]

Abbreviations:MD, mean diference; CI, confidence interval; SSRIs, Selective serotonin reuptake inhibitors; SNRIs, Serotonin and norepinephrine reuptake inhibitors.

#### 3.1.1 Selective Serotonin Reuptake Inhibitors (SSRIs)

SSRIs have been the most frequently recommended antidepressants, which may alleviate the symptoms of moderate to severe depression and are safer than other antidepressants. A meta-analysis suggested that concerning the effectiveness of SSRIs, fluoxetine and escitalopram with consistent efficacy in improving depression for adolescents compared with placebo (Hetrick et al., 2007). An additional trial revealed fluoxetine is not associated with an increase in suicidal ideation (Zhao et al., 2024). A subgroup analysis of the placebo-controlled clinical study resulted in significantly better efficacy of escitalopram than placebo in adolescents aged 12–17 years (Wagner et al., 2006). Therefore, the FDA approved fluoxetine for children aged 8 years and older, Escitalopram (Lexapro) for ages 12 years and older. Vilazodone is an antidepressant, which used to relieve MDD but is not approved for using in adolescents under 18. In the phase 3, double-blind, randomized controlled study in adolescents (12–17 years) with MDD to assess the efficacy, safety and tolerability of vilazodone, the outcome failed to demonstrate that vilazodone had an effect on MDD in adolescents (Durgam et al., 2018a). Monitoring symptoms and side effects closely after antidepressants administation is critical (Birmaher et al., 2007).

Improvements in depressive symptoms were significantly better in subjects who received fluoxetine in combination with cognitive behavioral therapy (CBT) and received fluoxetine alone compared with subjects who received placebo or CBT alone (March et al., 2007). A meta-analysis enrolling 71 trials (9510 participants) also showed that fluoxetine combined with CBT was more effective than CBT alone and psychodynamic therapy but not more effective than fluoxetine alone. In general, fluoxetine (alone or in combination with CBT) in adolescents with moderate to severe depression appears to be the optimum choice for acute treatment (Zhou et al., 2020), whereas SSRIs are not recommended for mild depression in adolescents (Wong et al., 2004), (Dudley et al., 2010).

#### 3.1.2 Serotonin Norepinephrine Reuptake Inhibitors (SNRIs)

SNRIs can relieve anxiety disorders and long-term (chronic) pain, of which recent evidence lacks support SNRIs as a primary treatment for adolescent depression (Jane Garland et al., 2016). A meta-analysis of clinical trials showed remission rates of 48.5% for SNRIs and 41.9% for SSRIs. The dropout rate due to poorly tolerated was higher in SNRIs than in SSRIs (Machado and Einarson, 2010). Venlafaxine is the representative drug of SNRI. Two placebo-controlled trials investigating the effectiveness of venlafaxine ER for the treatment of depression in adolescents found that it might be effective in depressed adolescents. As is a higher risk of suicide ideation compared to citalopram when applied to the adolescent population (Rubino et al., 2007), and the close monitoring is required during administration (Emslie et al., 2007). There is also insufficient evidence for the efficacy of duloxetine in adolescents (Emslie et al., 2014), (Atkinson et al., 2014).

#### 3.1.3 Tricyclics and others

Tricyclic antidepressants are unlikely to be beneficial for depression in adolescents and could also cause more vertigo, postural hypotension and thirst. An analysis including 12 studies concluded (Hazel et al., 2000), the lethality of tricyclic antidepressants in overdose and side effects implies their relatively little application in adolescents. A 2013 update of this study, which included 14 trials (590 participants), concluded that tricyclics are almost ineffective for adolescent depression. For this reason, tricyclic antidepressants are not recommended for pediatric or adolescent populations (Hazell and Mirzaie, 2013).

Bupropion for the treatment of MDD and seasonal affective disorder is an aminoketone antidepressant. It is practical and well-tolerated in adolescents suffering from depression. Side effects such as dizziness, insomnia, and loss of appetite may occur during treatment [(BC Mental Health, 2023)]. Generally, depressed adolescents taking bupropion for 2–3 months will experience improved depressive status (improved mood, better sleep, more energy, and improved quality of life). Where possible, including behavioral therapy such as interpersonal therapy (IPT) or CBT may be beneficial. But American Psychological Association (APA) clinical practice guideline only recommend bupropion for use in adults (Guideline Development Panel for the Treatment of Depressive Disorders, 2022).

Mirtazapine is a tetracyclic antidepressant with an obscure mechanism of action. It may be having a positive effect towards the communication among nerve cells in the central nervous system and/or restoring the chemical balance. Adult MDD is treatable with mirtazapine. Whether mirtazapine for MDD in adolescents is safe and effective is uncertain. An open-label, multiple centre study claims mirtazapine may be a valid method of treating major depression in adolescents, and common adverse events are fatigue, increased appetite and dizziness (Haapasalo-Pesu et al., 2004). However, the FDA has not approved mirtazapine for adolescents, and APA clinical practice guideline also against mirtazapine treatment for MDD in adolescents (World Health Organiztionc).

#### 3.1.4 Herbs - St. John’s wort

St. John’s is currently recommended for treating depression, menopausal symptoms, attention deficit hyperactivity disorder (ADHD) and other conditions. It is widely prescribed for depression in Europe. Early open-label studies suggested that St John’s Wort may be effective in mild and severe adolescent depression (Simeon et al., 2005), (Findling et al., 2003). However, St. John’s wort is known to interfere with the metabolism of some drugs and potentially causes serious side effects. Combining St. John’s wort with antidepressants may lead to elevated levels of serotonin, a brain chemical targeted by antidepressants, which is likely to be life-threatening. Based on the findings of previous surveys and drug properties, the guideline only recommend St John’s Wort for adults (World Health Organiztionc).

### 3.2 Integrative care

These can be referred to by the term “integrative care”, which primarily involve multidisciplinary care, for example “chronic care management, integrated behavioral healthcare, collaborative care”. Guidelines state that complex chronic diseases, such as depression, could be more successfully managed by proactive, patient-centered multidisciplinary care team[38]. Results for depressed adolescents from different primary care settings were significantly stronger than usual care in a 6-month study of Integrative Care [(Asarnow et al., 2005)]. For a randomized clinical trial followed by 12 months of integrative care as an intervention, with the Childhood Depression Rating Scale (CDRS-R; scale range, 14–94) as the outcome scale. Research shows that integrative care demonstrated stronger improvements in depressive symptoms at 12 months compared to conventional healthcare [(Richardson et al., 2014)]. Integrative care for adolescent depression could be integrated into primary care, according to the above.

### 3.3 Psychotherapy

Mild to moderate depression in adolescents with psychotherapy is the best treatment because it is effective and there is no risk of adverse effects or suicide. Psychotherapy combined with antidepressants can also improve the effectiveness of treatment. CBT and IPT-A are the major psychotherapeutic. We examined the efficacy of psychotherapy through a number of studies and meta-analyses.

#### 3.3.1 Cognitive behavioral therapy (CBT)

CBT is a psychosocial intervention designed to improve mental health. Originally used to treat depression, CBT has extended to treat many mental health conditions, including anxiety disorders. A meta-analysis (4335 participants) indicated the CBT is effective for young people with depression and as a preventive measure reduces the risk of depression by 63% (Bodden et al., 2019). Jesse B. Klein’s research also supports the effectiveness of CBT in adolescents (Klein et al., 2007). Most CBT studies with depressed adolescents found a reduction in suicidal ideation regardless of the form of CBT (i.e. individual, group) (Spirito et al., 2011). CBT is also combined with antidepressants for depression in adolescents, such as fluoxetine and venlafaxine, which is better than CBT alone, but no advantage over SSRIs alone [(Kutcher, 2008)]. Overall, the improvement of depression in adolescents with CBT, whether used alone or in combination with antidepressants, are supported by evidence of its safety and effectiveness.

The application of Digital Cognitive Behavioral Therapy (eCBT) is becoming more widespread. The traditional CBT is a face-to-face talking therapy that takes place in a direct one-to-one relationship between patient and therapist, and also allows team therapy (Luik et al., 2017). DCBT is a specific application that can be extended to email, video or other digital platforms. A review (n = 1575) found that dCBT was slightly superior to control group in improving depressive symptoms after early intervention, with no advantage at longer follow-up, and dCBT did not increase the risk of suicide (Ivlev et al., 2022). But it may improve insomnia among depressed patients (Cliffe et al., 2020).

#### 3.3.2 Adolescents with depression (IPT-A)

Interpersonal Psychotherapy for AIPT-A is an individual psychotherapy intended for adolescents between the ages of 12 and 18 (Weissman et al., 2003). The duration of IPT-A treatment is 12–16 weeks, and parents also receive some education about depression. IPT-A can improve the skill of cognitive, communication and problem-solving in adolescents; enhancing social functioning and reducing stress in interpersonal relationships, and it is effective for mild to moderate adolescent depression. IPT combine CBT with medication is the best choice for adolescents with moderate to severe depression (Vitiello, 2009).

#### 3.3.3 Family therapy

Negative family situations, family conflict, and other poor family relationships are high-risk factors for adolescent depression (Lewis et al., 2013). Family therapy is another model of psychotherapy that focuses on communication between the patient and family members to achieve therapeutic goals. It involves psychodynamic family therapy, structural family therapy, strategic family therapy, and cognitive behavioral family therapy (Broderick and Weston, 2009).The systemic family therapy available as a second-line treatment for depression and chronic illness is an effective intervention for adolescent (Cottrell and Boston, 2002).

#### 3.3.4 Neuroanalytic therapy

Influencing the patient’s behavior, thoughts, and feelings, neuroanalytic therapy is a talking therapy based on Sigmund Freud’s theory of psychoanalysis. This supports adolescents in controlling and managing their emotions. A retrospective study finds that neuroanalytic treatment relieves depressive symptoms and anxiety, and provides lasting effects (Hazell and Mirzaie, 2013). Another trial confirmed the effectiveness of neuroanalytic treatment in adolescents with severe mental disorders, with efficacy lasting up to 1 year and a remission rate of about 70% (Weitkamp et al., 2017).

### 3.4 Suicide risk management

Regular and thorough assessment of suicide risk is essential. This includes evaluating the severity of suicidal thoughts, the presence of a suicide plan, and the means available for suicide. It is crucial to collaborate with adolescents and their families to develop a safety plan, which should include identifying warning signs, implementing coping strategies, and listing emergency contacts. Adolescents at risk for suicide require close monitoring, particularly during the initial stages of treatment and when there are changes in medication or therapy. It is also essential to collaborate closely with mental health specialists and other healthcare providers to ensure comprehensive care and appropriate intervention when necessary.

### 3.5 Long-term monitoring

Scheduling regular follow-up appointments allows for ongoing assessment of the adolescent’s mental health status and treatment effectiveness.Continuously tracking symptoms of depression and any co-occurring conditions helps in adjusting treatment plans as needed. Evaluating adolescents’ functioning across various domains (e.g., school, social relationships, and family life) can provide insight into the overall impact of depression and its treatment. For adolescents on antidepressant medication, long-term monitoring includes assessing side effects, treatment adherence, and the need for dosage adjustments. Functional Assessment: Evaluating the adolescent’s functioning in various domains, such as school, social relationships, and family life, provides insight into the overall impact of depression and treatment effectiveness.

### 3.6 Relapse prevention

After achieving remission, continuation treatment for at least 6 months is recommended to prevent relapse. In some cases, maintenance treatment for longer periods may be necessary, especially for adolescents with a high risk of recurrence. Ongoing psychotherapy, such as CBT or IPT, can help adolescents develop coping skills and strategies to prevent relapse. Involving families in relapse prevention is also important. Educating families about depression, its symptoms, and how to support their adolescent can enhance the effectiveness of relapse prevention strategies. Additionally, teaching adolescents stress management techniques and promoting a healthy lifestyle can help reduce the risk of relapse.

## 4 Advances in the treatment of adolescent depression

Even though several therapeutic approaches are available to intervene with the depression in adolescents. However, The State of the World’s Adolescents 2021 report estimates 45,800 adolescent deaths from suicide globally each year (UNICEF, 2021). There are 828 antidepressants in development at the end of 2019 (Li et al., 2021), with novel targets such as N-methyl-D-aspartic acid receptor (NMDAR), dopamine receptor (DA), opioid receptor. Few antidepressants are under development for adolescents. However, antidepressants with a proven safety and efficacy profile will also be approved in adolescents. Table 5 lists the classification and mechanisms of some targets.

**TABLE 5:** New targets for antidepressant development.

Target	Representative drugs	Indication	Status	Sponsor
Non-competitive NMDAR antagonists	Axs-05	Major depression in adults	Phase 3	Axsome Therapeutics
	Spravato	Adult refractory depression	Approved	Johnson
An agonist for ADRβ3	Amibegron	Major depression in adults	Phase 3	Sanofi
Opioid receptor partial agonists	Buprenorphine/samidorphan	Refractory severe depression	Phase 3	Alkermes
NK1R receptor antagonists	L-759274	Major depression in adults	Phase 3	Merck
Selective OX2R antagonist	Seltorexant	MDD with insomnia symptoms	Phase 3	Johnson/minerva neurosciences
GABAAR positive allosteric modulator	Zuranolone	MDD and postpartum depression	Phase 3	Sage therapeutics
	Fengabine	MDD	Phase 3	Sanofi
Nicotinic channel modulator(nAChR)	Dexmecamylamine	Major depression	Phase 2	Targacept pharma, AZ
PDE4 inhibitors	Rolipram	Major depression	Phase 2	National Institute of Mental Health

Abbreviations: NMDA, N-methyl-D-aspartic acid receptor; ADRβ3, atypical beta3-adrenoceptors; NK1R, Neurokinin 1 (substance p) receptor; OX2R, orexin-2, receptors; GABAAR, γ-aminobutyric acid A receptor; nAChR, nicotinic acetylcholine receptor; PDE4, Phosphodiesterase-4; MDD, major depression disorder.

Clinical trials in phase 2 and phase 3 of the intervention for adolescent depression in Table 6 (Excluding completed trials), were screened from the “clinical trials ”database using the keyword “adolescent depression”.In addition to traditional psychological treatments, such as CBT and IPT-A, we found that there are other non-pharmacological treatment regimens, which include “Attachment-based family therapy”, “Primary Care Internet-Based Depression Prevention (CATCH-IT)”, “Transcranial Direct Current Stimulation (tDCS)”, “Attention Bias Modification Training (ABMT)”. The tDCS intervention widely tested for the treatment of major depression is a non-invasive technique that stimulates the prefrontal cortex and low-intensity continuous current changes cortical excitability, application to adolescents is safe and well-tolerated (Lee et al., 2017).

**TABLE 6:** Ongoing clinical trial of depression in adolescents.

Non-pharmacological			Antidepressants		
Intervention	No. of studies	Conditions	Intervention	No. of studies	Conditions
IPT-A	1	Depressive Disorder	Ketamine	5	Major Depressive, Suicide
CBT	3	Depressive	Omega-3 fatty acid	1	Depressive
Attachment-based family therapy	2	Depressive, Mood Disorder	Duloxetine hydrochloride	1	Depressive
Primary Care Internet-Based Depression Prevention	1	Depression, Anxiety Externalizing, Symptoms	Vortioxetine	1	Depressive
Transcranial Direct Current Stimulation	4	Major Depressive	Escitalopram	1	Major Depressive
Behavioral Activation Therapy	1	Depressive	N-acetylcysteine	1	Major Depressive
Attention Bias Modification Training	1	Major Depressive	Esketamine	1	Major Depressive
Family-Focused Treatment with MCC App	1	Mood Disorders Bipolar Disorder, Major Depression	Riluzole	2	Depression, And Bipolar Disorder, Anxiety Disorders

Note: From the “clinical trials” database, phase2-3, adolescents (0–17 years old).

We also identified some antidepressants and are trying to investigate their safety and tolerability in adolescents, such as ketamine, esketamine, N-acetylcysteine, and Riluzole. Ketamine is considered to be the most promising for adolescent depression, and studies have demonstrated that a monotherapy treatment can improve depressive symptoms for up to 2 weeks (Dwyer et al., 2021). Other promising anti-glutamate and antioxidant drugs such as riluzole and N-acetylcysteine are also being used for depression in adolescents. Previous studies have suggested the therapeutic potential of riluzole and N-acetylcysteine in adolescent depression, with a favorable safety profile (Fernandes et al., 2016), (Yao et al., 2020). Nevertheless, we are looking for more research to confirm both safety and efficacy in adolescents.

## 5 Recommendations and Conclusions

In conclusion, we have the following recommendations for depression treatment of adolescents: 1) Adolescents with mild to moderate depression are mostly treated with non-pharmacological treatment, examples are CBT and IPT-A; 2) Antidepressants are only a few approved for use in adolescents and require careful use under close monitoring; 3) Combined psychotherapy + SSRI therapy and Integrated Care are used to treat adolescents with major depression.

These treatments are all common and well-documented. A growing number of regimens for adolescent depression are being investigated, including some non-pharmacological therapies and antidepressants previously used in adults. Therefore, developing new interventions for adolescent depression holds some promise, and we are eagerly awaiting the introduction of new strategies.
